# Digital advantage in the COVID-19 response: perspective from Canada’s largest integrated digitalized healthcare system

**DOI:** 10.1038/s41746-020-00326-y

**Published:** 2020-08-31

**Authors:** Daniel C. Baumgart

**Affiliations:** Division of Gastroenterology, University of Alberta, Edmonton, AB Canada

**Keywords:** Health care, Information technology

## Abstract

The SARS-CoV-2 pandemic has challenged healthcare systems worldwide. Uncertainty of transmission, limitations of physical healthcare system infrastructure and supplies as well as workforce shortages require dynamic adaption of resource deployment to manage rapidly evolving care demands, ideally based on real time data for the entire population. Moreover, shut down of traditional face-to-face care infrastructure requires rapid deployment of virtual health care options to avoid collapse of health organizations. The Alberta Electronic Health Record Information System is one of the largest population based comprehensive electronic medical record (EMR) installations. Alberta’s long standing solid telehealth hardware-, training-, provider remuneration- and legislation infrastructure has enabled quick transition to virtual healthcare. Virtual health services including asynchronous secure clinical communications, real-time virtual care via messaging, telephony or video conferencing (telehealth) and ancillary functions like triage, scheduling, documentation and reporting, the previously established virtual hospital program with home monitoring, virtual health assessments, medication review, education and support for patients and families and coordination between family doctors, specialists and other health team members help to control viral transmission, protect healthcare personnel and save supplies. Moreover, rapid launch of online screening and triage tools to guide testing and isolation, online result sharing, infected patient and contact tracing including a smartphone exposure tracking application (ABTraceTogether), electronic best practice alerts and decision support tools, test and treatment order sets for standardized COVID-19 management, continuous access to population level real-time data to inform healthcare provider, public health and government decisions have become key factors in the management of a global crisis in Alberta.

## Introduction

The outbreak of novel pneumonia of probably bat origin^1^ in Wuhan China marked the beginning of a severe acute respiratory syndrome coronavirus 2 (SARS-CoV-2)^2^ pandemic^3^ that challenges healthcare systems worldwide. Uncertainty of transmission mode^4,5^, limitations of physical healthcare system infrastructure and supplies^6^ and workforce shortages^7,8^ require dynamic adaption of resource deployment to manage rapidly evolving care demands, ideally based on real time data for the entire population. Moreover, the social distancing^9^ based approach to flatten the disease-spread curve effectively shut down the traditional face-to-face care non-urgent delivery model aggravating the already existing overuse of emergency rooms and accelerating the collapse of health organizations.

Healthcare systems ahead of the digitalization curve, that have made the necessary investments into hardware-, training- and provider remuneration infrastructure prior to the pandemic can now enlist an important additional resource in their efforts to address the COVID-19 challenge.

## The healthcare system in Alberta

Alberta is home to more than 4.3 million people half of which live in two metropolitan areas, the Provincial capital Edmonton and the city of Calgary, whereas the remaining population spreads out over 600,000 square kilometers. All legal residents of Alberta are entitled to publicly funded and administered healthcare delivered by Alberta Health Services organized into five geographic zones. Alberta Health Services provides healthcare in 850 facilities of which 106 are acute care hospitals, five stand-alone psychiatric facilities, with 8,483 acute care beds, 27,163 continuing care beds/spaces, 249 community palliative and hospice beds and 2,772 addiction and mental health beds, the wholly-owned subsidiary Alberta Precision Laboratories plus equity partnership in 41 primary care networks. With 100,000 employees among them 8400 physicians Alberta Health Services is the largest employer in Alberta and fifth largest in Canada.

## The Alberta Electronic Health Record Information System (EHRIS)

The Alberta Electronic Health Record Information System^10^ dates back to 1997 and is jointly operated by Alberta’s Ministry of Health and Alberta Health Services. All available data items are catalogued in the Alberta Health Data Asset Directory and the Alberta Health Services Data Asset Inventory Summary^11,12^ Its main domains comprise access tools, repositories, registries, and infrastructure. *Access Tools* (i.e. the Alberta Netcare Portal^13^) allow users to browse and view health data across repositories, but hold no actual patient data. *Repositories* capture, store and maintain patient diagnostic, treatment, and care information. *Registries* capture, store, and provide data on places, identities, and events used by repositories and access tools for identification purposes. *Infrastructure* finally yet importantly, enables connection and transmission of health data.

Connect Care was launched in November 2019^14^ to implement the vision of one-patient-one-record. It is the central access point for comprehensive health information and delivery for patients and providers and replaced 1300 independent health information systems across the province. Connect Care^15^ is based on a highly customized version of EpiCare (Epic Inc., WI, USA) to meet the needs of Alberta and Albertans. Wave 1 was rolled out in the Edmonton Zone and the remaining waves are expected to follow between 2020 and 2022^16^.

## Digital advantage during the COVID-19 response

### Self-assessment online tool for citizens

Alberta Health Services quickly developed a self-assessment tool to help citizens in Alberta determine whether they need to be tested, direct them to the appropriate test center and advise on self-isolation^17^.

### Self-assessment online tool for healthcare & shelter workers, enforcement personnel and first responders

Healthcare workers, group home-, disability support- and shelter workers, police-, peace-, environmental health- and fish-and wildlife officers, correctional facility staff as well as service personnel in hospitals, clinics, and pharmacies are all at an increased risk. This targeted tool helps to prioritize groups at highest risk of local exposure and at-risk populations. It determines if testing, additional advice and/or self-isolation are required^18^.

### Fit for work online assessment for healthcare workers

An additional online questionnaire was developed to determine fitness for work before arriving at the workplace so efficiency and exposure risk at local screening stations are reduced with screening personnel^19^.

### Linking online screening with testing, online result sharing and tracing

Through the Alberta Electronic Health Record Information System^10^ SARS-CoV-2 test results are available to all providers and patients in Alberta. The online screening tool data above is linked to the SARS-CoV-2 test results via the Alberta Health Care Number. If an Albertan tests positive for COVID-19, a contact tracer from Alberta Health Services gets in touch with the infected person using contact information provided at the testing center to determine others who may have been exposed to the infected person over the previous 21 days^20^ and encourage enrollment into the app enabled digital tracing program (see below).

### Standardized management with best practice advisories, pandemic data collection and decision support

The Connect Care system contains rapidly developed best practice alert and decision support modules that prompt providers to consider SARS-CoV-2 testing based on entered clinical data (i.e. medical history, symptoms, diagnoses) or test result data (i.e. diagnostic imaging, laboratory test results). Providers are also asked to collect additional pandemic relevant data with structured questionnaires that links this information to the online tool screening tool data and test results if the patient underwent such screening prior to presentation to the healthcare system. This helps to refine the online test tools and later also study of the impact of the online screening. Moreover, Connect Care provides newly developed and continuously improved standardized diagnostic and treatment order sets for patients with suspected or established SARS-CoV-2 infections and COVID-19. This is particularly relevant for advanced pandemic stages where care has to be provided by medical professionals not primarily trained in internal medicine, pulmonology or critical care.

### Alberta trace together mobile device software application

The Government of Alberta also deployed a mobile exposure contact tracing application^20,21^. It is based on mobile devices exchange of Bluetooth enabled secure encrypted tokens when another device with the app installed is detected nearby. Upon interaction with a contact tracer (see above) app users are encouraged to voluntarily upload the encrypted data from the app to Alberta Health Services and allow using that information to reach and warn other app users who have been within approximately 2 meters as determined by received signal strength indicator (RSSI) readings.^20^

### Telemedicine and digital healthcare

It was estimated that between two and three million virtual visits took place between Canadians and their clinicians in 2019^22^. The Alberta telemedicine standard of practice dates back to 2010^23^. Advice bulletins on electronic communications & security of mobile devices^24^ and telemedicine^25^ have been kept up-to-date for more than a decade. With the advent of the SARS-CoV-2 pandemic professional bodies such as Alberta’s College of Physicians and Surgeons^26^ and the Alberta Medical Association^27^ provided additional advice and training while the Canadian Medical Protective Association covered medical legal aspects^28^ and cyber security^29^.

Virtual health services including asynchronous secure clinical communications, real-time virtual care via messaging, telephony or video conferencing (telehealth) and the ancillary functions like triage, scheduling, documentation and reporting help control viral transmission, protect healthcare personnel and save supplies.

Alberta Health Services operates several secure clinical communication channels including the Connect Care Secure Chat messaging system for patients and providers that is directly integrated into the electronic medical record and Secure Email.

Real-Time virtual care is facilitated through integration of Zoom (Zoom Video Communications, Inc., San Jose, CA, USA) technology with Connect Care. Zoom recording and live streaming for social media functions have been disabled.

This enables electronic referrals through the online Alberta Referral Directory (including from referring providers outside the Connect Care environment), electronic triage, electronic visits, electronic consultations, video visits, and other virtual services that take advantage of the enterprise scheduling, decision-support, documentation and reporting capabilities of a fully integrated clinical information system. In addition, other conventional telehealth suites like Skype for Business (Microsoft Inc. Redmond, WA, USA) and Real Presence (Poly Inc., Santa Cruz, CA, USA) are available. All technology is in compliance with the College of Physicians and Surgeons of Alberta guidelines on secure electronic communication and acceptable to the Office of the Privacy Commissioner of Alberta.

Lastly, patients in Alberta can access many elements (e.g. test results, medications, immunizations) of their own medical record online either through MyHealth Records in Netcare or MyChart in Connect Care.

### Virtual clinical hospital meetings and multidisciplinary conferences

Due to the existing digital infrastructure, we were able to switch all clinical meetings like morning report, hand-over, weekend sign-out, radiology and rounds, but also our multidisciplinary conferences for transplant-, cancer- or inflammatory bowel disease patients within 24 h.

### Virtual hospital and other care in the community programs

Alberta Health Services has previously invested into enhancing community care^30^, which now pays off. The Edmonton Virtual Hospital for instance can deliver acute care in the home for individuals living with chronic or complex diseases provided by physicians, pharmacists, nurses and paramedics. This is accomplished through home monitoring, virtual health assessments, medication review, education and support for patients and families and coordination between family doctors, specialist doctors and other health team members. It can shorten hospital stays or avoid them all together, which reduces the risk of SARS-CoV-2 transmission for these often-frail patients and their providers.

### Additional billing codes for telehealth released

In addition to the existing telehealth billing codes, the Alberta Ministry of Health has made amendments to the Schedule of Medical Benefits^31^ to support increased facilitation of service delivery through virtual means^32^.

## Additional benefits of a digitally connected healthcare system during a crisis

A networked integrated healthcare system adds to, or perhaps fully enables the digital advantage. Critical data like case volume, ICU and regular bed capacity at facility-, city-, and are level can be tracked across an entire population without insurance-, health management organization- or institutional boundaries. Emergencies can be managed through a central command structure. Task forces for personal protective equipment, infection, prevention and control and supply management are easily appointed. Critical information can be shared in real time with the entire healthcare system., Policies are implemented everywhere and disaster planning can make optimal use of existing resources including deployment of medical staff across different facilities with city- and zone wide service and backup service schedules as well as privilege extensions as they are ultimately provided by a single payer.

## Future impact and challenges of digital healthcare in the world

### Pandemic driven acceleration of healthcare digitalization

The SARS-CoV-2 pandemic accelerates the digital revolution^33^ in healthcare, which began some 15 years ago^34^. Through this crisis we are rethinking our traditional doctor-patient relationship, some elements of which will evolve towards a virtual care provider^35^. Of course, the successful management of a global health crisis does not exclusively depend on the digitalization level of the healthcare system. However, highly digitalized healthcare systems employing networked electronic medical record systems with real time data collection, result sharing and decision support tools, can improve active surveillance, timely case finding, effective isolation, testing, tracing of every contact in containment and enforce social distancing, all of which have been identified by the WHO Strategic and Technical Advisory Group for Infectious Hazards as key factors to control the COVID-19 pandemic^36^.

### Data security, privacy and ethical concerns

Rising concerns of cyber security with broad financially driven exploitation of personal data by tech companies, criminal infiltration of networked infrastructure and government systems, repeated major data breaches in our increasingly digitally connected world have led jurisdictions like the European Union to pass legislation for tighter regulation (General Data Protection Regulation) and better protection of citizens’ right to informational self-determination. The legal^37,38^ and ethical^39^ challenge is to balance^40^ individual’s interest in privacy^41,42^ with the societal need to use large volumes of digital health and mobility data^43^ to successfully take on the pandemic challenge^44,45^. Government overreach^46^ or criminal abuse of programming loop holes in increasingly deployed tracing apps, especially in non-democratic countries are a real concern. Another prominent example are the encryption flaws in the widely used Zoom video conferencing software (Zoom Video Communications, Inc., San Jose, CA, USA) and transmission of meeting encryption keys through China, exposed by the University of Toronto in April 2020^47^.

### Not all economies are equally prepared for digitalization in healthcare

Whereas governments and health system operators in many Western jurisdictions will hopefully expedite building and expanding a digital medicine infrastructure^48^ along with the required training, human resources and investment in academic research, to further refine it, the hurdles for countries with a lower United Nations Human Development Index (HDI)^49^ are often insurmountable, considering that some parts of the world are still in the process of building basic technical infrastructure like functional electric grids or internet connectivity to accommodate such systems.
